# Exploring the Multifunctional Role of *Paenibacillus* Metabolites in Various Fields

**DOI:** 10.3390/ijms262412089

**Published:** 2025-12-16

**Authors:** Panhong Yuan, Zonghui Song, Langjie Zhang, Zhengsheng Pan, Xiaolong Chen

**Affiliations:** College of Biotechnology and Bioengineering, Zhejiang University of Technology, Hangzhou 310014, China

**Keywords:** *Paenibacillus*, biological characteristics, applications, challenges

## Abstract

*Paenibacillus* has attracted considerable scientific and practical attention in recent years owing to its diverse biological characteristics and extensive range of applications. Its applicability spans multiple fields, including agriculture, medicine, and industrial biotechnology. However, the widespread utilization of *Paenibacillus* is hindered by several challenges, such as environmental variability, biosafety concerns, and technical barriers. Current research efforts are increasingly directed toward elucidating the diversity and mechanisms of action of its bioactive metabolites. This review offers a comprehensive synthesis of the current state of knowledge on *Paenibacillus*, covering its metabolic capabilities, practical applications across various sectors, existing limitations, and prospective avenues for future research.

## 1. Introduction

*Paenibacillus* has garnered considerable scientific interest in recent years owing to its diverse biological characteristics and broad spectrum of applications [1,2]. Advances in molecular identification methods, particularly 16S rRNA gene sequence analysis, have significantly enhanced the understanding of its taxonomic classification, facilitating more precise and reliable species delineation [3]. These Gram-positive, rod-shaped bacteria have unique morphological and physiological traits, including distinctive spore formation and specific flagellation patterns. These traits are critical for their environmental persistence and functional adaptability across diverse ecological niches [4].

The physiological and biochemical characteristics of *Paenibacillus* are highly remarkable. Its nutritional versatility, optimal growth conditions across temperature and pH ranges, and capacity to synthesize a diverse array of metabolites—including enzymes and antimicrobial compounds—underscore its metabolic flexibility [5]. Genomic investigations have further elucidated the genetic foundations of these traits, revealing key gene clusters associated with critical functions such as antimicrobial peptide biosynthesis and the regulation of spore formation [6]. The metabolic products of *Paenibacillus* display exceptional diversity. Antimicrobial agents, such as polymyxins and other antimicrobial peptides, specifically target structural components of pathogenic organisms, whereas hydrolytic enzymes like chitinases and cellulases play pivotal roles in plant disease suppression [6]. Volatile organic compounds (VOCs) and additional secondary metabolites contribute significantly to plant growth promotion and the modulation of host-microbe interactions.

*Paenibacillus* exhibits a broad ecological distribution, inhabiting diverse natural environments such as soil, plant rhizospheres, and extreme habitats [7]. Its capacity to establish symbiotic associations with various host plants, coupled with its ability to withstand a wide range of environmental stresses through sophisticated adaptive mechanisms—including endospore formation and stress-responsive regulatory pathways—highlights its significant ecological role.

The applications of *Paenibacillus* span a wide range of fields. In agriculture, it functions as both a biocontrol agent and a plant growth promoter, contributing to sustainable agricultural practices [8]. In medicine, polymyxins play a critical role in the treatment of multidrug-resistant Gram-negative bacterial infections, and ongoing research is focused on developing safer and more effective derivatives [9]. In industrial biotechnology, extracellular enzymes and optimized fermentation processes for the production of antimicrobial peptides provide promising biotechnological tools [10]. Nevertheless, the broad-scale application of *Paenibacillus* encounters several challenges. Environmental variability can compromise the efficacy of bacterial formulations, while safety concerns—such as the nephrotoxicity associated with polymyxins and the potential for horizontal gene transfer of resistance genes—must be rigorously evaluated [11]. Additionally, technical barriers, including limited efficiency of current gene editing systems and an incomplete understanding of the regulatory mechanisms governing metabolic pathways, continue to impede further advancement [12].

This review aims to provide a comprehensive synthesis of the current understanding of *Paenibacillus*, encompassing its taxonomic and functional diversity, the mechanisms of action of its metabolites, ecological distribution, and its applications across various fields. By identifying key challenges and outlining future research priorities, this article seeks to establish a solid foundation for the continued exploration and effective utilization of these versatile bacteria.

## 2. The Diversity and Mechanism of Action of Metabolites Produced by *Paenibacillus*

**Figure 1:**
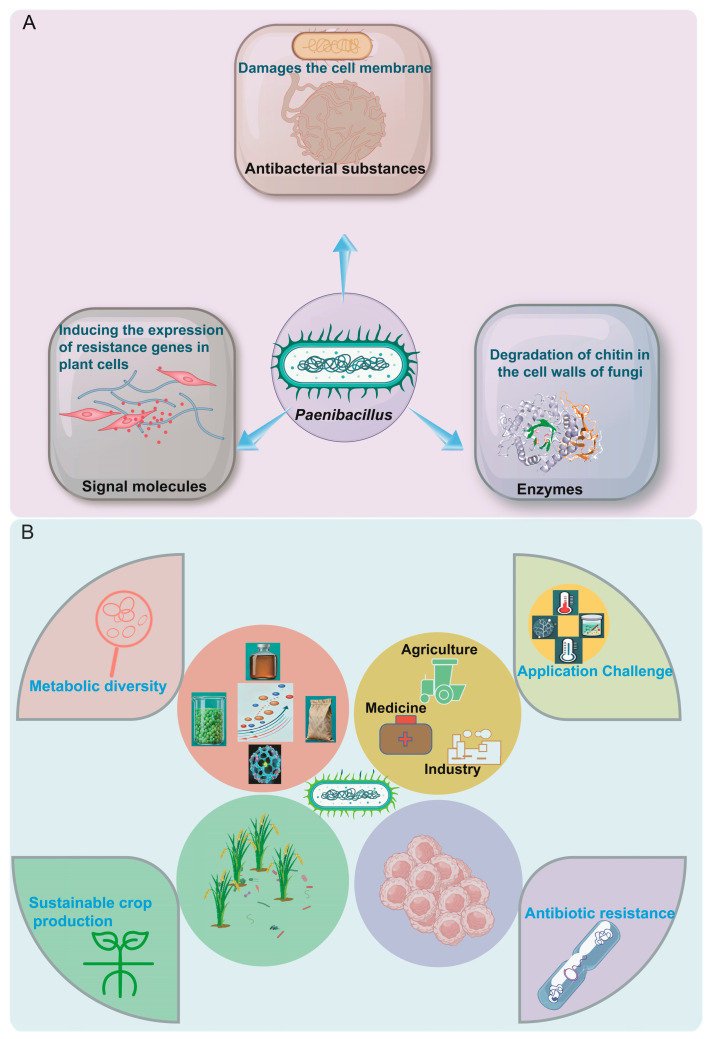
(**A**) Functional attributes and biological roles of *Paenibacillus*. (**B**) Diversity and applications of the genus *Paenibacillus*. Metabolic diversity encompasses antibiotic synthesis and resistance-related metabolism, antibacterial activity-associated metabolic pathways, and the biosynthesis and function of novel antifungal agents. These diversified metabolic products are leveraged for practical applications in agriculture, medicine, and biotechnology.

The ecological dynamics of antibiotic resistance encompass both the extensive evolutionary history of antibiotic synthesis in natural environments and the recent emergence of resistance traits in pathogenic microorganisms driven by anthropogenic antibiotic use. Central components of the resistome include intrinsic resistance genes present in environmental bacteria, which have gradually formed and accumulated over millennia, as well as their capacity for horizontal gene transfer—enabling the dissemination of resistance among disease-causing microorganisms (Figure 1). Understanding the mechanisms by which resistance traits evolve and spread across diverse bacterial taxa is essential for addressing the growing public health threat posed by multidrug-resistant organisms. Research has revealed evidence of an ancient and intrinsic resistome within *Paenibacillus* sp. LC231, a bacterial strain isolated from the remote and pristine Lechuguilla Cave ecosystem. Through biochemical assays and transcriptomic profiling, scientists have investigated the resistance determinants of *Brevibacillus brevis* VM4, the second characterized species within the *Paenibacillaceae* family, known for its production of diverse antimicrobial secondary metabolites. Comprehensive phylogenomic analyses further demonstrate that the resistomes of the *Paenibacillaceae* lineage are undergoing continuous evolution, largely independent of secondary metabolite biosynthetic pathways, and are characterized by the presence of concealed, duplicated, pseudo-paralogous, and conserved orthologous genes. Evidence indicates that, unlike in pathogenic species, mobile genetic elements play a limited role in reshaping the resistome, suggesting divergent evolutionary trajectories in resistance mechanism development between pathogens and environmental bacteria [13].

*Paenibacillus* sp. UY79 exhibited suppressive activity against a range of fungal and oomycete pathogens assessed in this study, with agar-diffusible substances and volatile organic compounds contributing to its antagonistic effects. Co-application of strain UY79 with rhizobia did not impair symbiotic establishment or alfalfa plant growth promotion. Genome-wide analysis identified several genes potentially involved in biocontrol-related functions within the UY79 genome. Furthermore, the results from genomic exploration and antimicrobial assays suggest that UY79 may modulate the growth of bacterial communities commonly found in soil and plant-associated environments [14]. The analysis and characterization of naturally occurring congeneric compounds provide valuable insights into the relationship between chemical structure and biological function. A novel antifungal agent, KB425796-A, has been isolated from the recently identified bacterial strain *Paenibacillus* sp. 530603. Among its analogs, KB425796-C demonstrated significant antifungal activity against various micafungin-resistant fungal pathogens. These findings indicate that KB425796-C holds promise as a candidate for the development of therapeutic agents targeting micafungin-resistant fungal infections [15]. The strain *P. polymyxa* KM2501-1 produces a spectrum of VOCs with demonstrated nematicidal activity, among which 2-undecanol stands out as a potential biocontrol agent targeting *Meloidogyne incognita*. Despite its efficacy, the exact mechanism by which 2-undecanol prevents root invasion by root-knot nematodes remains poorly understood. Laboratory tests reveal that 2-undecanol displays diverse anti-nematode activities in vitro, including contact toxicity, fumigation effects, attraction behavior, inhibition of locomotion—evidenced by 84.0% and 97.0% reductions in head thrashing and body bending frequency at 40 mg/L—and nearly complete suppression of egg hatching. Treatment with 2-undecanol resulted in a marked decline in gall development on roots, with a control rate of 60.8% achieved at a dosage of 5 mg per pot. Metabolomic analysis showed that exposure to 2-undecanol significantly reshapes the composition of tomato root exudates. Among 17 differentially accumulated metabolites, the elevated levels of 10-undecenal were found to attract and subsequently kill *M. incognita* J2 juveniles at 100 mg/L, whereas cyclohexylamine displayed nematicidal properties at 1000 mg/L. These results imply that 2-undecanol has potential as a sustainable strategy for managing plant-parasitic nematodes [16]. The bacterium *P. larvae* has been found to produce a variety of non-ribosomal peptides (NRPs) as well as hybrid secondary metabolites that integrate peptide and polyketide structural elements (NRP/PK). Within this array of compounds, lipopeptides structurally resembling iturins—named paenilarvins A–C—have been identified. Iturin-family lipopeptides are widely acknowledged for their strong antifungal activity, and some representatives have demonstrated cytotoxicity toward mammalian erythrocytes and various human cancer cell lines in previous studies [17].

There are ribosomally synthesized peptides such as paenibacillin, paenilan, and paenicidin [18]. The first group of substances is produced independently of RNA, with their presence determined by genes encoding lipopeptide synthetases, among other factors [19]. The most common antifungal lipopeptide of the genus *Paenibacillus* is fusaricidin [20]. Fusaricidin is composed of hexapeptide rings containing at least one bond in addition to the amide bonds, with an attached guanidinylated ß-hydroxy fatty acid [21]. Biosynthesis of fusaricidins is encoded by the fus gene cluster, which consists of eight genes—*fusA*, *fusB*, *fusC*, *fusD*, *fusE*, *fusF*, *fusG*, *fusH*—with the *fusA* gene being crucial as it encodes the protein involved in the synthesis of the main structure of the substance, making it the most important gene in the cluster [22]. Fusaricidins A, B, C, and D exhibit considerable activity against fungi by causing damage to the membrane structure, but show only marginal activity against Gram-negative bacteria [23].

## 3. Research on the Application of *Paenibacillus* in Different Fields

### 3.1. Application of Paenibacillus in the Field of Agriculture

**Figure 2:**
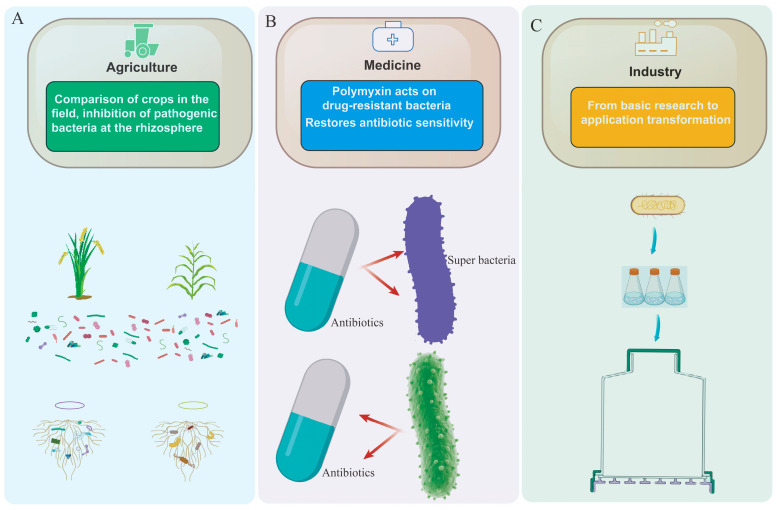
Comparison of the applications and efficacy of *Paenibacillus* across sectors. (**A**) Agricultural applications of *Paenibacillus*. (**B**) Medical applications of *Paenibacillus*. (**C**) Industrial applications of *Paenibacillus*.

Prolonged and excessive use of chemical bactericides for managing bacterial infections in crops has resulted in several adverse consequences, including ecological disruption, persistent toxic residues, and the growing resistance of pathogenic bacteria to these agents (Figure 2). In response, numerous studies have focused on developing biological control strategies as environmentally sustainable alternatives to conventional chemical treatments. Le et al. examined the antimicrobial properties of the fermented supernatant derived from *Paenibacillus elgii* JCK-5075, along with its active components, assessing their efficacy against plant-pathogenic bacteria using both in vitro and in vivo experimental systems. Four pelgipeptin compounds—designated A, B, C, and D—were isolated from *P. elgii* JCK-5075 and exhibited strong, broad-spectrum antibacterial activity [24]. Polyketides and lipopeptides produced by *Bacillus* and *Paenibacillus* species have been widely recognized for their potent antimicrobial effects. These bioactive compounds hold significant promise as eco-friendly, naturally sourced agents for controlling human pathogens in clinical contexts and mitigating plant diseases in agricultural settings [19]. Apple replant disease (ARD) represents a major constraint on the sustainable development of the apple industry. The application of biological control methods offers a viable and environmentally responsible approach to alleviating the impact of ARD. In this context, a bacterial strain, *Paenibacillus polymyxa* GRY-11, was isolated from the rhizosphere soil of healthy apple trees in established orchards. Experimental findings demonstrated that this strain exhibits substantial antifungal activity against key fungal pathogens associated with ARD, achieving inhibition rates of 80.00%, 71.60%, 75.00%, and 70.00% against *Fusarium moniliforme*, *F. proliferatum*, *F. solani*, and *F. oxysporum*, respectively. These results highlight the potential of *P. polymyxa* GRY-11 as an effective microbial agent for the biological control of ARD [25]. *Atractylodes chinensis* is a valuable medicinal plant extensively utilized across East Asia; however, its cultivation is frequently hindered by root rot, a disease that significantly reduces both yield and quality. From the rhizosphere of healthy *Atractylodes chinensis* plants, 68 antagonistic bacterial strains were isolated. Among them, strain SY42 displayed the highest antifungal efficacy, with inhibition rates of 67.07%, 63.40%, and 68.45% against *F. oxysporum*, *F. solani*, and *F. redolens*, respectively. Based on morphological characterization and molecular identification, SY42 was classified as *P. polymyxa*. Furthermore, the volatile organic compounds emitted by SY42 were found to effectively suppress mycelial growth of pathogenic fungi through a diffusion-mediated mechanism [26].

The findings demonstrated that *P. polymyxa* Sx3 significantly inhibited the growth of 20 distinct *Xanthomonas oryzae pv. oryzae* strains. Studies using rice seedlings revealed that *P. polymyxa* Sx3 not only enhanced plant growth but also conferred resistance to bacterial leaf blight. Furthermore, microbiological analyses confirmed that *P. polymyxa* Sx3 possesses the capacity to fix atmospheric nitrogen, solubilize phosphate, and produce indole-3-acetic acid, suggesting that multiple mechanisms contribute to its plant growth-promoting properties. In addition, the liquid culture of *P. polymyxa* Sx3 was shown to inhibit bacterial proliferation, suppress biofilm formation, and alter the cellular morphology of the *Xoo* strain GZ0005 [27]. *Paenibacillus mucilaginosus* is widely recognized as a plant growth-promoting rhizobacterium (PGPR). This strain exhibits multiple beneficial traits, including suppression of plant pathogens, biofilm formation, phosphate solubilization, and synthesis of indole-3-acetic acid. Genomic analysis identified 26 gene clusters associated with secondary metabolite biosynthesis, while genetic characterization indicated potential resistance to antibiotics such as ampicillin, bacitracin, polymyxin, and chloramphenicol [28]. Microbial volatile organic compounds (MVOCs) have garnered significant attention due to their ability to promote plant growth. *Paenibacillus peoriae*, a strain isolated from mangrove rhizosphere soil, produces VOCs that enhance the growth of *Arabidopsis thaliana* seedlings, increase above-ground biomass, and stimulate lateral root development in *A. thaliana*. Moreover, *P. peoriae* is capable of synthesizing both volatile and soluble metabolites that not only promote plant growth but also confer protection against pathogens, highlighting its potential for application in sustainable agricultural systems [29]. *P. polymyxa* is a well-characterized rhizobacterium known for its plant growth-promoting capabilities and substantial potential in biological disease control. Wheat sheath blight, caused by the soilborne fungal pathogen *Rhizoctonia cerealis*, is a severe disease that markedly reduces wheat yields, prompting extensive research into biological control strategies. *P. polymyxa* ZYPP18 exhibits strong in vitro antagonistic activity against *R. cerealis* and effectively suppresses the progression of sheath blight in excised wheat leaves. Research has shown that ZYPP18 promotes plant growth and possesses the ability to solubilize phosphate and synthesize indole-3-acetic acid. These findings suggest that *P. polymyxa* ZYPP18 is a promising microbial agent with dual functionality in enhancing plant development and managing plant diseases [30]. Collectively, these studies deepen our understanding of the growth-promoting mechanisms of PGPR strains and underscore their significant potential for use in agriculture as sustainable bioinoculants.

### 3.2. Application of Paenibacillus in the Field of Medicine

Polymyxins are antibiotics first discovered in 1947 during research aimed at identifying agents effective against Gram-negative bacterial infections. They are produced by the Gram-positive bacterium *P. polymyxa* and are characterized by a cyclic peptide core attached to a fatty acyl moiety. Their primary bactericidal mechanism involves disruption of the bacterial cell membrane. To date, two clinically utilized variants—polymyxin B and colistin—have been developed and are commonly employed as last-resort therapies for infections caused by multidrug-resistant pathogens [31,32]. Antimicrobial resistance has emerged as a critical global public health threat. Among existing antibiotics, carbapenem β-lactams are often considered the final therapeutic option for severe infections caused by multidrug-resistant bacterial strains. Colistin, a cationic polypeptide antibiotic, is frequently deployed as the ultimate line of defense against carbapenem-resistant bacteria. A newly identified plasmid-mediated colistin resistance gene, *mcr-2*, was detected shortly after the discovery of the prototype *mcr-1* gene, which has since disseminated globally. Evolutionary analyses indicate that MCR-1 and MCR-2 likely originated from a phosphoethanolamine transferase homolog involved in lipid A modification found in *Paenibacillus*, a genus known for producing polymyxins. Transcriptomic studies have demonstrated that *mcr-2* exhibits higher expression levels compared to *mcr-1*. Furthermore, gene knockout experiments have confirmed that the transmembrane domains of both MCR-1 and MCR-2 are essential for their proper localization and functional activity within the bacterial periplasm [33]. The transmembrane domain of the MCR protein is a key structural element for its stable anchoring on the bacterial cell membrane. This protein modifies the chemical structure of the cell membrane lipid A, reducing its surface negative charge, thereby weakening the ability of polymyxins to bind to it and conferring resistance to such antibiotics in the bacteria. If the transmembrane domain is absent, the MCR protein will not be able to be localized on the cell membrane, and thus lose its ability to modify lipid A, resulting in the complete failure of the resistance function [34]. Moreover, the transmembrane domain is highly hydrophobic and can effectively match the lipid bilayer environment of the cell membrane, thereby maintaining the structural stability of the protein on the membrane. Even under environmental stress conditions such as temperature fluctuations and osmotic pressure changes, this structure can still ensure the correct localization and functional integrity of the MCR protein [33]. Therefore, the transmembrane domain is not only the basis for the biological function of the MCR protein but also an important molecular mechanism for bacteria to cope with various environmental stresses, including antibiotic pressure.

The increasing prevalence of multidrug-resistant bacterial strains has placed a substantial burden on healthcare systems and underscored the urgent need for novel classes of antibiotics. Bacterial lipopeptides are secondary metabolites typically synthesized by nonribosomal peptide synthetases, which frequently display broad-spectrum antimicrobial activity. Over the past four decades, only two new structural classes of antibiotics—linezolid and the lipopeptide daptomycin—have been approved for clinical use. While numerous bacterial species are capable of producing lipopeptides, certain genera, particularly *Bacillus* and *Paenibacillus*, have demonstrated exceptional capacity in producing structurally diverse and potent antimicrobial agents. Several of these compounds have been known for decades and may serve as promising candidates for the discovery and development of next-generation antibiotics [35].

### 3.3. Application of Paenibacillus in the Industrial Field

*Paenibacillus* sp. strain FPU-7 is recognized as a highly efficient chitin-degrading microorganism. Chitin and its derivatives hold significant potential for applications across diverse industries, including medicine, agriculture, and food processing. In addition to producing extracellular chitinases, this bacterial strain employs a specialized multimodular chitinase enzyme, ChiW, which plays a crucial role in the degradation of chitin into oligosaccharides at the cell surface. These oligosaccharides are subsequently internalized and further metabolized by β-N-acetylhexosaminidase in the cytoplasm, ultimately yielding N-acetyl-D-glucosamine [36]. A novel alginate lyase belonging to the polysaccharide lyase family PL-31 was identified and characterized from *Paenibacillus ehimensis*. The enzyme was successfully secreted extracellularly in *E. coli* and displayed substrate specificity toward poly β-D-mannuronate. When sodium alginate was used as the substrate, the enzyme exhibited peak catalytic activity at pH 7.5 and 55 °C in the presence of 50 mM NaCl. Due to its exceptional thermostability and catalytic efficiency, *P. ehimensis* shows strong potential for the industrial-scale production of alginate oligosaccharides [37].

The incorporation of *P. mucilaginosus* into the composting process extended the thermophilic phase, resulting in enhanced degradation of organic matter and more efficient transformation of nitrogenous compounds. Furthermore, the application of this bacterial strain significantly altered the microbial community structure during both the initial heating and thermophilic stages. The use of *P. mucilaginosus* in industrial-scale composting demonstrates considerable potential as a viable strategy to improve nutrient cycling and mitigate the dissemination of antibiotic resistance genes [10]. *P. polymyxa*, has attracted substantial attention due to its agricultural applications, functioning both as a PGPR and as an effective biocontrol agent. Strain HY96-2, isolated from the tomato rhizosphere, was successfully developed into the world’s first microbial biopesticide based on this species for the control of plant diseases. This breakthrough has facilitated its large-scale commercialization within China [38].

The application of *Paenibacillus* in composting is mainly achieved in the form of a composite microbial agent, often in combination with *Bacillus subtilis*, *Bacillus amyloliquefaciens*, and *Pseudomonas*. This is mainly because the composite microbial agent can significantly enhance the efficiency and stability of composting through functional complementation and ecological niche collaboration [39]. The technology has been widely applied in the collaborative composting of sludge and agricultural waste, high-nitrogen composting of livestock manure, and lignin degradation composting of garden waste. The industrial-scale production scale of *Paenibacillus* microbial agents is closely related to the downstream composting process and raw material processing capacity. In industry, large-scale cultivation is usually carried out using a liquid deep fermentation process. The volume of the fermentation tank varies from 10 m^3^ to over 100 m^3^ depending on the enterprise scale. During the composting process, the mineralization of organic nutrients and effective retention of inorganic nutrients occur. In the terms of carbon cycle, it promotes the degradation of organic matter and promotes the formation of humus; in the nitrogen cycle, it supplements nitrogen sources through nitrogen fixation and regulates urease activity to reduce ammonia volatilization, achieving efficient retention and transformation of nitrogen [40]. In addition, the spores formed by *Paenibacillus* during the high-temperature stage of composting have strong heat resistance and stress resistance. They can germinate and colonize in the rhizosphere environment after being applied to the soil, continuously exerting nitrogen fixation, phosphorus solubilization, and potassium release functions, thereby extending the nutrient benefits of compost to the soil-plant system and constructing an integrated sustainable nutrient cycling chain of “compost-soil-plant”.

## 4. The Research Challenges and Future Prospects of *Paenibacillus*

### 4.1. The Research Challenges of Paenibacillus

The production strain DSM 33618 was derived from a *Paenibacillus lentus* strain previously evaluated by the European Food Safety Authority (EFSA) and confirmed to be safe. The genetic modifications introduced into the strain pose no safety concerns, and no antibiotic resistance genes associated with the modification were detected in the final production strain. No viable cells or residual genetic material from the production strain were detected in the product used during the formulation of the additive. Endo-1,4-β-d-mannanase (Hemicell^®^ HT/HT-L), produced by *P. lentus* DSM 33618, is considered safe for target animal species when administered under the intended conditions of use [41]. The application of this feed additive does not present any risk to human health or the environment. It is non-irritating to skin and eyes; however, it may induce allergic reactions upon dermal contact and has the potential to cause respiratory sensitization. The additive has demonstrated efficacy at a dosage of 32,000 U/kg in broiler chickens, laying hens, minor poultry species raised for meat or for reproduction and egg production, growing pigs, and minor porcine species [41]. American Foulbrood (AFB) is a severe and highly contagious bacterial disease affecting honey bee brood, resulting in significant colony losses worldwide. The causative agent, *Paenibacillus larvae*, is a Gram-positive bacterium capable of infecting bee larvae within the first few days of development. This pathogen is globally prevalent in apiaries, and its spores can remain viable for extended periods. Conventional antibiotic treatments have limited effectiveness in controlling AFB, as they are only active against the vegetative bacterial stage. Once clinical signs of infection are observed in a hive, the only scientifically validated method to prevent further transmission is complete incineration of the hive, associated equipment, and the entire bee colony. Due to its aggressive nature and substantial threat to apiculture, AFB is classified as a notifiable disease on a global scale. Consequently, there is an urgent need for safe, effective, and environmentally sustainable strategies to protect the health and stability of honey bee populations [42].

Bacterial contamination poses a dual threat, not only compromising human health through pathogenic infection but also undermining the quality of milk and its processed derivatives by promoting spoilage. Of particular concern are aerobic spore-forming bacteria, including species from the genera *Sporosarcina*, *Paenisporosarcina*, *Brevibacillus*, *Paenibacillus*, *Geobacillus*, and *Bacillus*. These microorganisms present significant challenges due to their high resistance to industrial pasteurization processes and their capacity to form biofilms within piping systems and stainless steel equipment. Regardless of whether they consist of single or multiple microbial species, such biofilms serve as persistent reservoirs of spoilage organisms, potentially initiating recurring cycles of contamination. Extensive research has confirmed that these bacteria frequently colonize critical sites in dairy processing environments, such as pipe terminations, corners, fissures, gaps, seals, valve assemblies, and equipment joints. Therefore, the implementation of effective monitoring and intervention strategies is essential to mitigate spoilage risks and protect public health. Conventional control measures typically include clean-in-place protocols, the application of chemical or biological antimicrobial agents, and the investigation of innovative, advanced control technologies [43]. Ropy spoilage in bread is characterized by a sticky, fibrous disintegration of the crumb structure, accompanied by the formation of slimy exudates, discoloration, and an odor reminiscent of decaying fruit. The growing consumer preference for preservative-free products, coupled with the impacts of global climate change, may increase the prevalence of this spoilage type. Several bacterial species have been identified as causative agents of bread ropiness, including *B. amyloliquefaciens*, *B. subtilis*, *B. licheniformis*, members of the *B. cereus* group, *B. pumilus*, *B. sonorensis*, *Cytobacillus firmus*, *Niallia circulans*, *P. polymyxa*, and *Priestia megaterium*. Maintaining process hygiene alone is insufficient to prevent contamination, as flour is inherently susceptible to colonization by these *Bacillus* species. This susceptibility arises from their natural occurrence as part of the endogenous microbial flora of wheat and their ability to produce heat-resistant endospores, which remain viable throughout processing, baking, and storage [44].

Due to the thick cell wall structure and low genetic transformation efficiency in spore-forming bacteria, the gene editing efficiency of the CRISPR-Cas9 system in the genus *Paenibacillus* remains limited at approximately 20% [10]. Furthermore, the molecular interaction mechanism between the key sporulation regulator Spo0A and secondary metabolite synthetases has not yet been fully elucidated, thereby hindering precise engineering of the antifungal lipopeptide biosynthesis pathway. When scaling up fermentation from a 5 L to a 500 L bioreactor, spore yield decreases by approximately 30%, primarily due to limitations in oxygen mass transfer efficiency. Concurrently, the high dependence on complex nitrogen sources results in a significant increase of about 40% in raw material costs. To date, the long-term ecological risks of fusaricidin on non-target soil microorganisms remain insufficiently assessed. To address these technical challenges, there is an urgent need to develop efficient, marker-free genome editing tools and tightly regulated inducible promoter systems to enable fine-tuned control of key metabolic pathways [45]. Additionally, integration of multi-omics datasets—including transcriptomics and metabolomics—is essential for constructing comprehensive gene regulatory network models [46]. Advancing the synergistic integration of synthetic biology with bioprocess engineering will be critical. Implementing fed-batch fermentation processes under real-time pH and dissolved oxygen (DO) feedback control can facilitate scalable production while maintaining consistent product performance.

### 4.2. The Future Prospects of Paenibacillus

Although limited progress has been achieved, several research gaps remain. The antimicrobial mechanisms of *Paenibacillus* are not yet fully elucidated, particularly under real-world agricultural conditions. While numerous bioactive compounds have been identified, it remains unclear whether these substances act directly against phytopathogens, induce systemic resistance in plants, or confer protection through alternative mechanisms. For *Paenibacillus* and related strains, assessing their impact on indigenous microbial communities and genetic profiles is essential, as such evaluations can yield valuable insights into the modes of action of plant growth-promoting bacteria used in agricultural applications. Moreover, examining the interactions between *Paenibacillus* and other beneficial microorganisms, including fungi, may uncover synergistic relationships that enhance both plant protection and growth.

*Paenibacillus* species are widely recognized for their capacity to promote plant growth, with numerous strains demonstrating the ability to enhance nutrient uptake in plants, suppress phytopathogens, and synthesize plant hormones. Although the application of *Paenibacillus* as a bioinoculant in agricultural systems may be constrained by various environmental factors, ongoing research on their adaptation and functional performance within complex soil ecosystems holds promise for facilitating broader adoption as biofertilizers. Beyond their agricultural applications, these bacteria are also capable of producing a diverse array of bioactive compounds, including antimicrobial agents, enzymes, which have significant implications for medical therapies, industrial biotechnology, and environmental remediation, with certain products already commercialized. However, not all effects associated with this genus are beneficial, as some species have been implicated in food spoilage, bee diseases, and sporadic human infections. Despite these limitations, continued investigation and optimization are expected to unlock additional potential, enabling *Paenibacillus* to contribute meaningfully to human health and sustainable development [9].

## 5. Conclusions

*Paenibacillus* has garnered growing scientific interest due to its remarkable metabolic versatility and ability to synthesize a diverse range of bioactive compounds. Beyond its function in pathogen suppression, this genus demonstrates substantial biotechnological promise through its capacity to promote plant growth, produce industrially valuable enzymes, and serve as an effective biofermentation platform. In summary, *Paenibacillus* represents a highly promising resource for advancing sustainable agriculture and biotechnological innovation. Future research should focus on closing the current knowledge gaps, optimizing large-scale production methods, and developing integrated strategies for its practical deployment in real-world applications. Fully harnessing its functional potential could establish *Paenibacillus* as a cornerstone in the development of environmentally sustainable solutions across agricultural, industrial, and related sectors.

## Data Availability

No new data were created or analyzed in this study. Data sharing is not applicable to this article.
